# The Combination of Human Urinary Kallidinogenase and Mild Hypothermia Protects Adult Rats Against Hypoxic-Ischemic Encephalopathy-Induced Injury by Promoting Angiogenesis and Regeneration

**DOI:** 10.3389/fnagi.2018.00196

**Published:** 2018-07-11

**Authors:** Xiaoya Gao, Haiting Xie, Shuzhen Zhu, Bin Yu, Ying Xian, Qian Ouyang, Yabin Ji, Xiaohua Yang, Chunyan Wen, Penghua Wang, Yufeng Tong, Qing Wang

**Affiliations:** ^1^Department of Neurology, Zhujiang Hospital of Southern Medical University, Guangzhou, China; ^2^Department of Rehabilitation, Zhujiang Hospital, Southern Medical University, Guangzhou, China; ^3^Department of General Intensive Care Unit of Lingnan Hospital, The Third Affiliated Hospital of Sun Yat-sen University, Guangzhou, China; ^4^Department of Neurosurgery, Zhujiang Hospital, Southern Medical University, Guangzhou, China; ^5^Department of Neurology, Nanfang Hospital, Southern Medical University, Guangzhou, China; ^6^Department of Microbiology & Immunology, School of Medicine, New York Medical College, Valhalla, NY, United States; ^7^Structural Genomics Consortium, Department of Pharmacology and Toxicology, University of Toronto, Toronto, ON, Canada

**Keywords:** hypoxic-ischemic encephalopathy, neuroprotectant, human urinary kallidinogenase, mild hypothermia, angiogenesis, regeneration

## Abstract

**Objectives**: Human Urinary Kallidinogenase (HUK) is a tissue kallikrein that plays neuroprotective role in ischemic conditions via different mechanisms. Mild hypothermia (MH) is another robust neuroprotectant that reduces mortality but does not profoundly ameliorate the neurological outcome in hypoxic-ischemic encephalopathy (HIE) patients. However, whether the combination of HUK and MH can be used as a promising neuroprotective treatment in HIE is unknown.

**Methods**: One-hundred and forty-four adult Wistar rats were randomly divided into five groups: Sham, HIE, HUK, MH and a combination of HUK and MH treatment. The HIE rat model was established by right carotid dissection followed by hypoxia aspiration. The survival curve was created within 7 days, and the neurological severity scores (NSS) were assessed at days 0, 1, 3, and 7. Nissl staining, Terminal deoxynucleotidyl transferase-mediated dUTP nick end-labeling (TUNEL), immunofluorescent staining and western blotting were used to evaluate neuronal survival, apoptosis and necrosis, tight-junction proteins Claudin-1 and Zonula occludens-1 (ZO-1), vascular endothelial growth factor (VEGF), doublecortex (DCX), bradykinin receptor B1 (BDKRB1), BDKRB2 and Ki67 staining.

**Results**: The combined treatment rescued all HIE rats from death and had a best survival curve compared to HIE. The Combination also reduced the NSS scores after HIE at days 7, better than HUK or MH alone. The combination of HUK and MH reserved more cells in Nissl staining and inhibited neuronal apoptosis and necrosis as well as significantly attenuated HIE-induced decreases in claudin-1, ZO-1, cyclin D1 and BDKRB1/B2 in comparison to HUK or MH treatment alone. Moreover, the combined treatment increased the expression of VEGF and DCX as well as the number of Ki67-labeled cells.

**Conclusions**: This study demonstrates that both HUK and MH are neuroprotective after HIE insult; however, the combined therapy with HUK and MH enhanced the efficiency and efficacy of either therapy alone in the treatment of HIE, at least partially by promoting angiogenesis and regeneration and rescuing tight-junction loss. The combination of HUK and MH seems to be a feasible and promising clinical strategy to alleviate cerebral injury following HIE insult.

**Highlights**:

- The combination of HUK and MH distinctly reduces neurological dysfunction in HIE rats.
- HUK enhances the neuroprotective effects of MH in HIE.
- MH attenuates tight-junction disruption, upregulates the BDKR B1/2, DCX and cyclin D1.
- The combination of MH and HUK enhances the expressions of MH/HUK mediated-BDKR B1/2, DCX, cyclin D1 and Ki67 positive cells.

## Introduction

Hypoxic-ischemic encephalopathy (HIE) is a quite common and severe disorder in neonates, children and adults and is also a leading cause of mortality and morbidity. The neuro-pathogenesis of brain injury in patients with HIE remains largely elusive. The molecular mechanisms may include brain-blood-barrier (BBB) breakdown, tight junction and microvascular disruptions, edema and cell death (Guardia Clausi et al., 2016; Kossatz et al., 2016; Thornton et al., 2017). A better understanding of the pathogenesis of HIE is needed, and it is necessary to develop various novel and effective treatments for HIE brain injury.

It has been documented that mild hypothermia (MH) is effective in the treatment of HIE. The potential mechanisms of MH may include the dramatic inhibition of autophagosomes and lysosomes; reduction of the activity of neuronal nitric oxide synthase (nNOS) and apoptotic caspase enzymes (Wood et al., 2016); downregulated expression of lectin-like oxidized low-density lipoprotein receptor-1 (LOX-1), tumor necrosis factor-α (TNF-α) and oxidant species (Akamatsu et al., 2014); and induction of cold-shock proteins and heat shock proteins correlated with subsequent apoptosis (Lee et al., 2017). Clinically, although therapeutic MH alleviates cell damages and results in lower death rates in HIE (Mitra et al., 2017), the limited efficacy of hypothermia is insufficient and only slightly improves serious neurological impairments (Shankaran et al., 2012; Manley et al., 2017). Therefore, it is necessary to find a strategy to enhance the neuroprotective effects of MH in the treatment of HIE. These facts also inspired us to explore combined treatments of MH with certain neuroprotectants. In our previous study, we screened a series of medicines that might have synergistic effects or enhance the positive effects of MH (34°C for 4.5 h) on primary cortical neurons following oxygen glucose deprived (OGD/R) treatment and identified the potential neuroprotectant human urinary kallidinogenase (HUK; Gao et al., 2014, 2016). Whether the combination of HUK and MH could also provide enhancing effects in the HIE adult model is of interest.

HUK is a tissue kallikrein originally isolated from human urine that cleaves low molecular weight kininogen to release bradykinin (Stankowski and Gupta, 2011). HUK can mediate a complex series of physiological actions through its receptor signaling pathways, and suppress inflammatory mediators such as interleukin-1(IL-1), interleukin-6 (IL-6), or TNF-*α*, selectin E (ELAM-1), monocyte chemoattractant protein-1 (MCP-1), and Nuclear Factor-κB (NF-κB; Chen et al., 2010); increase erythrocyte deformability; prolong the re-calcification time; lower blood viscosity (Chao and Chao, 2006); and inhibit platelet aggregation, apoptosis, hypertrophy and fibrosis (Chao et al., 2010). Accumulated clinical evidence has suggested that HUK is an effective therapy for acute ischemic stroke (Zhang et al., 2012; Li et al., 2015; Miao et al., 2016; Ni et al., 2017). Recent studies by Han and Li indicated that HUK promotes post-ischemic angiogenesis and cerebral perfusion via induction of vascular endothelial growth factor (VEGF) and activation of bradykinin B1/B2 receptors (BDKRB1/B2), which further enhanced the expression of VEGF (Han et al., 2015; Li et al., 2015). In spite of the similar pathophysiological characteristics of HIE and ischemic stroke, both of which are caused by low blood and oxygen support and have ischemic penumbra (Gao et al., 2014), few studies have shown how HUK functions under HIE insult and whether HUK functions via mediating VEGF and BDKRB1/B2 receptors. Since VEGF and doublecortex (DCX) are usually taken as the indices of angiogenesis and neurogenesis respectively, in this study we used them as the markers of angiogenesis and neurogenesis (Couillard-Despres et al., 2005; Garcia et al., 2014; Zhao et al., 2017). Whether HUK exerts beneficial effects on neurons under HIE insult and how the combination of HUK and MH are synergistic or enhance positive effects need to be explored.

Therefore, in the present study, we sought to determine the following: (1) whether HUK enhances the efficacy of MH in terms of the rat survival rate, cell death, tight junction preservation and restoration of neurological function in the HIE adult rat model; (2) how the expression of VEGF and BDKRB1/B2 receptors mediates the additional protection of combined treatment; and (3) whether the combination of HUK and MH provides enhanced neurogenesis by regulating the expression of DCX and Ki67 compared to either treatment alone in HIE adult rats.

## Materials and Methods

### Ethics Statement

The experiments in this study were approved and carried out in accordance with the Institutional Animal Care and Use Committee of the Laboratory Animals Center, Southern Medical University. The protocol was approved by the Institutional Animal Care and Use Committee (Animal Ethic Approval No: NYFF-2015-87). We certify that the rats in our study were treated in accordance with the National Institutes of Health Guide for the Care and Use of Laboratory Animals (NIH Publications No. 80-23) revised 1996 guidelines. We attest that all efforts were made to minimize the number of animals used and their suffering.

### Experimental Design

**Figure 1:**
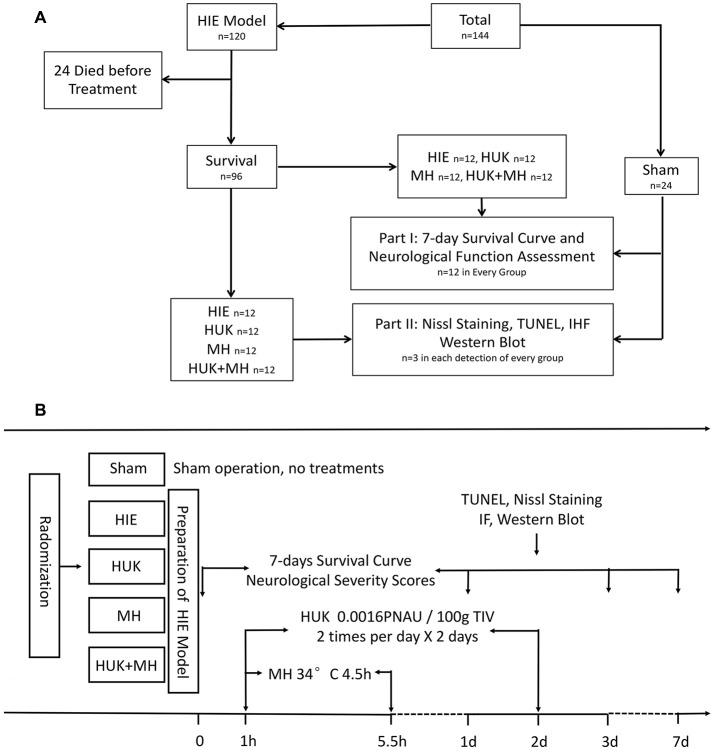
Division and treatment of animals. **(A)** The total profile of the animals which were subjected to the experiment. A total of 144 adult male Wistar rats were used in this experiment. The rats were randomly divided into five groups: Sham, Hypoxic-ischemic encephalopathy (HIE), Human urinary kallidinogenase (HUK), Mild hypothermia (MH), and Combination group (*n* = 24). Before treatment, 24 rats died after HIE was induced, and eight died after treatment, two in HUK group, two in MH group and four in HIE group. The 7-day survival curve and neurological function assessment (neurological severity scores, NSS) were performed within 7 days (*n* = 12). Rats were euthanized and detected at the 48 h after HIE (*n* = 3 for every detection). **(B)** The division, treatments and time sequence of the experiment. The rats were randomly divided into five groups: Sham, HIE, HUK, MH and HUK + MH (combination) group. Rats in Sham group accepted sham operation and no treatments. Rats in HIE received operation with no treatment; rats in HUK were injected with 0.0016 PNAU/100 g HUK via the tail vein twice a day in successive 2 days, starting from 1 h after inducing HIE; rats in the MH group were treated with MH (34°C) for 4.5 h starting at 1 h after inducing HIE; and rats in the combined treatment group (HUK+MH) accepted HUK and MH treatment as prescribed. The 7-day survival curve and neurological function assessments (at day 0, 1, 3, 7) were performed. Terminal deoxynucleotidyl transferase-mediated dUTP nick end-labeling (TUNEL), Nissl, immunofluorescent staining and Western Blot were performed at the 48-h time point after HIE.

A total of 144 adult male Wistar rats weighing 300–350 g were obtained from the Experimental Animal Center of Southern Medical University. The rats were randomly divided into five groups: Sham group, which underwent a sham operation; HIE group, which was subjected to right carotid dissection followed by hypoxia aspiration (8% O_2_ with 92% N_2_ for 3 h; Edwards et al., 2017) to simulate a medium to severe hypoxia-ischemic encephalopathy neurological severity scores (NSS; 7–18 scores); HUK group, which was injected with 0.0016 PNAU/100 g HUK (Techpool Bio-pharma, Guangzhou, China) via the tail vein twice a day in successive 2 days, starting from 1 h after inducing HIE; MH group, which was treated with MH (34°C) for 4.5 h started from 1 h after inducing HIE; and combined treatment group (HUK+MH), which was administered with HUK and MH treatment as preceding methods. The 7-day survival curve and NSS (at days 0, 1, 3, 7) were determined to evaluate the effectiveness of HIE model. Nine rats in each group were euthanized, the brains were obtained from rats at 48 h after HIE to do the paraffin sections and were used for assessment using Nissl, Terminal deoxynucleotidyl transferase-mediated dUTP nick end-labeling (TUNEL), immunofluorescent staining and Western Blotting (Figure 1).

### HIE Animal Model

The Wistar adult male rats were anesthetized using 5% isoflurane and maintained under 1.5% isoflurane anesthesia with 70% N_2_ and 30% O_2_, retaining a left femoral artery intubation while monitoring the rectal temperature. HIE model was set up and modified following previous studies (Harding et al., 2016; Yang et al., 2016; Edwards et al., 2017). Briefly, the neck hair was shaved, and the skin was disinfected first. Then a 15 mm-long incision was made above the sternum and at the midline of the neck. Next, the subcutaneous tissue, sternohyoid and sternocleidomastoid muscles were bluntly separated. When the right common carotid artery pulse could be seen, we dissociated the common carotid artery and its two branches of the internal and external carotid artery, ligated the proximal and distal end of the internal carotid artery and mutilated the internal carotid artery in the middle of the two ligations. After confirming no bleeding, we finally sutured the muscles and skin layer by layer and disinfected the incision. Afterward, the rats were placed into a chamber under 8% hypoxia with 92% N2 inhalation for 3 h while maintaining the rectal temperature at 38°C with a thermostatic pad. By using Transcranial Doppler (TCD) to verify that there was no blood flow in the right carotid, the model was considered to be successfully established.

### 7-day Survival Curves and Neurological Severity Scores (NSS)

Seven-day survival curves were made according to the time and quantity of the rat deaths in each group. Neurologic deficiency was assessed on the basis of modified NSS assessment. The NSS evaluation consisted of motor (0–6 scores), sensory (0–2 scores), reflex (0–4), and balance (0–6 scores) tests, with the results measured on a scale of 0–18. Score 0 indicates normal neurologic function, score 1–6 indicates mild deficiency, score 7–12 indicates moderate deficiency, score 13–18 indicates severe deficiency (score 18 indicates death). All the mice subjected to neurological severity assessments were evaluated by three blinded, trained investigators after inducing HIE (day 0) and were reassessed at days 1, 3, and 7.

### Terminal Deoxynucleotidyl Transferase-Mediated dUTP Nick End-Labeling (TUNEL) Staining

Five sections of the dentate gyrus (DG) of right hippocampa in each rat were detected. In each section, five areas were randomly chosen to analyze. TUNEL staining was conducted using the *in situ* Cell Death Detection Kit (Roche, 11684795910, Germany) following the manufacturer’s instructions. Briefly, we prepared 5 μm thick paraffin sections that were then immersed in dimethylbenzene I and II (Sigma Aldrich, 296333, USA) for 15 min and then sequentially dewaxed at 100%, 85%, 75% ethanol (Sigma Aldrich, 49836, USA) and distilled water for 5 min. Next, we incubated the sections with diluted protease K (DAKO, Cat. #S3020, USA) at 37°C for 30 min and with Permeabilization Buffer (Aviva, OOMB00004, USA) for 20 min at room temperature. Finally, we incubated the sections with a mixed TUNEL working solution (TdT: dUTP = 2:29) at 37°C for 2 h, stained the nuclei with DAPI (Sigma Aldrich, D9542, USA) at room temperature for 10 min, and sealed the sections with the anti-fluorescence quenching sealing agent Fluoroshield™ (Sigma Aldrich, F6182, USA). The prepared sections were photographed with a NIKON ECLIPSE TI-SR inverted fluorescence microscope (NIKON, Japan) with a UV excitation wavelength 330–380 nm, emission wavelength 420 nm (for DAPI); and FITC excitation 465–495 nm, emission 515–555 nm (for TUNEL). The images were quantified using Image-Pro Plus 6.0 software (Media Cybernetics Inc., Rockville, MD, USA). The ratio of the number of TUNEL-positive cells to the number of total nuclei (DAPI staining) was used to present the intensity of the TUNEL.

### Nissl Staining

Nissl staining was used to observe neurons in the right hippocampal dentate gyrus (DG) of the insulted hemisphere with Toluidine Blue Staining Reagent (Sigma Aldrich, 89640, USA). Briefly, we dewaxed the paraffin sections (as for TUNEL staining) and stained them with 5% toluidine blue at room temperature for 10 min. Sections were then soaked in 95% ethanol (Sigma Aldrich, 49836, USA) for 2 min, dimethylbenzene (Sigma Aldrich, 296333, USA) for 3 min, and sealed with neutral balsam (Thermo Fisher Scientific Inc., B10100, USA). The Nissl staining slides were observed using a Nikon microscope (Nikon, Eclipse Ci-E, Japan). We used neuronal number per mm^2^ to measure the survival neurons in Nissl staining. We counted the number of the neurons in 50 × 50 μm^2^ of the initial images, then calculated the average neuron number per mm^2^.

### Immunofluorescence

Five sections of the DG regions of each hippocampus were processed for the immunofluorescence detection. Immunofluorescence detection was conducted at 48 h after HIE. The paraffin sections were put into dimethylbenzene I and II (Sigma Aldrich, 296333, USA) for 15 min and then sequentially dewaxed at 100%, 85%, 75% ethanol (Sigma Aldrich, 49836, USA) and distilled water for 5 min, respectively. For antigen retrieval, we incubated the sections with citrate buffer antigen retrieval solution (pH 6.0; Sigma Aldrich, C9999, USA) in a microwave oven on medium-temperature heat for 8 min and medium-low-temperature heat for 7 min. After blocking with a 5% BSA (Sigma Aldrich, B2064, USA) solution for 1 h, we incubated the sections with primary antibody, anti-VEGF (a marker of angiogenesis, mainly in vascular endothelial cells; 1:1000, AbCam, ab46154, UK), anti-doublecortin (a marker of neurogenesis; 1:1000, AbCam, ab18723, UK), or anti-Ki67 (a non-specific proliferation of cells; 1:1000, AbCam, ab15580, UK) antibody for 2 h at room temperature. After washes, the sections were incubated with secondary antibody, goat anti-rabbit IgG (Cy3; 1:5000, AbCam, ab6939, UK) or goat anti-rabbit IgG (FITC; 1:5000, AbCam, ab6717, UK) for 1 h, and then DAPI (Sigma Aldrich, D9542, USA) at room temperature for 5 min. Finally, the sections were sealed with the anti-fluorescence quenching sealing agent Fluoroshield™ (Sigma Aldrich, F6182, USA). The images were collected with a NIKON Fluorescent Microscope (NIKON, ECLIPSE C1, Japan) at excitation wavelength 330–380 nm and emission wavelength 420 nm (blue); FITC excitation 465–495 nm (green) and emission 515–555 nm; and CY3 excitation 510–560 nm and emission 590 nm (red). The images were analyzed using Image-pro plus 6.0 (Media Cybernetics Inc., Rockville, MD, USA). Five areas were randomly chosen to analyze in each image. The signal densities of VEGF, DCX and Ki67 were normalized by the density of the signals of nuclei.

### Western Blotting

For Western Blot analysis, rats were euthanized at 48 h after HIE and all the right hippocampal tissue was collected for total protein extraction. The concentration of protein was determined using the BCA method with the Pierce™ BCA Protein Assay Kit (Thermo Fisher Scientific Inc., 23225, USA). Thirty-five microgram of protein from each sample was separated using 4%-12% sodium dodecyl sulfate-polyacrylamide gel electrophoresis (SDS-PAGE), and then the proteins were transferred to PVDF membranes (Millipore, IPFL00010, USA). After blocking with 5% BSA (Sigma Aldrich, B2064, USA) for 1 h, the membranes were incubated with the primary antibodies anti-claudin-1 (1:1000, AbCam, ab15098, UK), anti-Zonula Occludens (anti-ZO-1; 1:1000, AbCam, ab96587, UK), anti-BDKRB1 (1:1000, Bioss, bs-8675R, USA), anti-BDKRB2 (1:1000, Novus Biologicals, NBP1-46328, USA), anti-cyclin D1 (1:1000, AbCam, ab16667, UK), and anti-GAPDH (1:5000, AbCam, ab8245, UK) at 4°C overnight. After three washes, the membranes were incubated with the secondary antibody Goat Anti-Rabbit IgG H&L (HRP; 1:5000, AbCam, ab6721, UK) or antibody Goat Anti-Mouse IgG H&L (HRP; 1:5000, AbCam, ab6789, UK) at room temperature for 1 h. The bands were developed using Luminant Western HRP Substrates (Millipore-Sigma, WBLUM0100, Germany) and exposed using X-ray photography (Kodak, USA). Gray value (GV) analyses for the quantification of the bands were performed using Quantity One Analysis Software (Bio-Rad Laboratories, Quantity One 1-D, USA). Relative GVs of protein bands were normalized to the internal control GAPDH.

### Statistical Analyses

Continuous data are presented as the mean ± standard deviation (SD). For the survival curve, Log-Rank Test (Kaplan-Meier) was used. For the repeated measurement data, Mauchly’s test of sphericity was used to determine whether they fit the spherical distribution. When the data did not fit the spherical distribution, the MANOVA test was made first, followed by LSD test being used to compare multiple groups. For the two-factor design, two-way ANOVA was used to analyze the interaction of two factors, and the main or single effect of each factor was analyzed by a General Linear Model. For multiple comparisons, analyses were conducted by two-way ANOVA followed by Tukey’s test. The significance level was set at *P* < 0.05, two-tailed. All statistical analyses were performed using IBM SPSS Version 20 (SPSS Statistics V24, IBM Corporation, Armonk, NY, USA).

## Results

### The Combination of HUK and MH Improved the Survival Curve and Increased the Neurological Function

**Figure 2:**
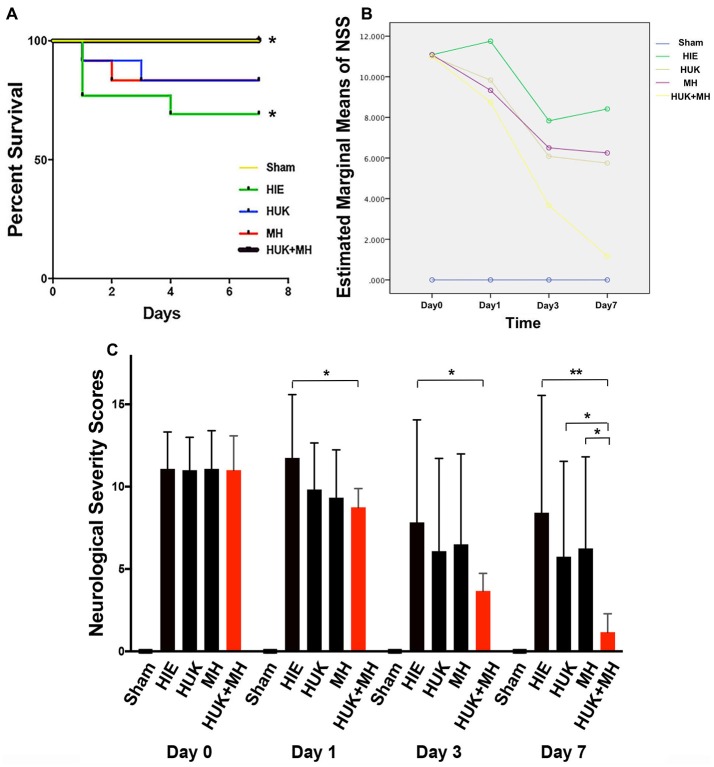
Survival curves and the analysis of NSS. **(A)** 7-day survival curves: the 7-day survival curves were created according to the deaths in each group at different time points (*n* = 12). The survival curve of HIE group was the lowest in the survival curve chart, 4 out of 12 died, three were at day 1 and one at day 4. HUK or MH ended with two deaths of 12. The best survival curve (0 out of 12 died) was noted when HUK was combined with MH, which was the same as the Sham Group (0 out of 12 died). The survival curves of HUK and MH groups have no difference with that of the HIE group (Log-Rank Test (Kaplan-Meier), χ2 = 0.848, *P* = 0.357), but the survival curve of combination is better than that of the HIE group (Log-Rank Test (Kaplan-Meier), *χ*^2^ = 4.615, *P = 0.032). **(B)** The analysis of NSS scores: Mauchly’s test of sphericity shows the NSS scores does not fit the spherical distribution (Mauchly’s test of sphericity, *χ*^2^ = 85.722, ***P < 0.001). There is an interaction between the different treatments and the time points (MANOVA, *F* = 6.990, ***P < 0.001). **(C)** NSS analyses in groups: among the Sham, HIE, HUK, MH and combination of HUK and MH groups (*n* = 12), NSS assessments were carried out at day 0, day 1, day 3 and day 7. The bar shows the NSS score of every group. The NSS scores are statistically different at different time points (MANOVA, *F* = 61.585, ***P < 0.001). At day 0, there is no statistical difference among different treatments (MANOVA, *F*_day 0_ = 0.006, *P*_day 0_ = 0.999). At day 1 and day 3, only combination, not HUK or MH alone reduced the NSS scores after HIE (MANOVA, LSD test, *P_day 1: Combi-HIE_ = 0.013, *P_day 3: Combi-HIE_ = 0.049). At day 7, the combination reduces the NSS scores compared to HIE (MANOVA, LSD test, **P_Combi-HIE_ = 0.002), and better than HUK or MH (MANOVA, LSD test, *P_Combi-HUK_ = 0.043, *P_Combi-MH_ = 0.026). But neither HUK nor MH were different to the HIE group (MANOVA, LSD test, *P*_HUK-HIE_ = 0.233, *P*_MH-HIE_ = 0.331). *P < 0.05, **P < 0.01, ***P < 0.001.

The survival curve of HIE group was the lowest in the survival curve chart, 4 out of 12 rats died, and three were at day 1 and one at day 4 (Figure 2A). HUK or MH ended with 2 deaths among 12 rats but had no difference of survival with the HIE group (Log-Rank Test (Kaplan-Meier), *χ*^2^ = 0.848, *P* = 0.357, Figure 2A). The best survival (0 out of 12 died, Figure 2A) was noted when HUK was combined with MH, which was the same as the Sham Group and better than the HIE group (0 out of 12 died, Log-Rank Test (Kaplan-Meier), *χ*^2^ = 4.615, *P = 0.032, Figure 2A).

For NSS analysis, the Mauchly’s test of sphericity was used to confirm that the NSS did not fit the spherical distribution (Mauchly’s test of sphericity, *χ*^2^ = 85.722, *P* < 0.001). There is an interaction between the different treatments and the time points (MANOVA, *F* = 6.990, *P* < 0.001, Figure 2B). Among the Sham, HIE, HUK, MH and combination of HUK and MH groups (*n* = 12), the NSS scores are statistically different (MANOVA, *F* = 61.585, ***P < 0.001). At day 0, there is no statistical difference among different treatments (MANOVA, LSD test, *F*_day 0_ = 0.006, *P*_day 0_ = 0.999, Figure 2C). At day 1 and day 3, only the combination of HUK and MH, but not HUK or MH alone reduced the NSS scores after HIE (Combination_day 1_: 8.750 ± 1.138, HIE_ day1_: 11.750 ± 3.841, MANOVA, LSD test, *P_day 1: Combi-HIE_ = 0.013; Combination_day 3_: 3.667 ± 1.073, HIE_day 3_: 7.833 ± 6.221, MANOVA, LSD test, *P_day 3: Combi-HIE_ = 0.049, Figure 2C). At day 7, there are statistical difference among different treatments (MANOVA, *F*_day 7_ = 3.828, *P_day 7_ = 0.016, Figure 2C). The combination reduces the NSS scores compared to HIE (Combination: 1.167 ± 1.115, HIE: 8.417 ± 7.128, MANOVA, LSD test, **P_Combi-HIE_ = 0.002, Figure 2C), and better than HUK or MH (HUK: 5.750 ± 5.786, MH: 6.250 ± 5.562, MANOVA, LSD test, *P_Combi-HUK_ = 0.043, *P_Combi-MH_ = 0.026, Figure 2C). However, neither HUK nor MH were different to the HIE group (MANOVA, LSD test, *P*_HUK-HIE_ = 0.233, *P*_MH-HIE_ = 0.331, Figure 2C).

### HUK Increased the Survival Neurons, and the Combination of HUK and MH Provided More Neuroprotection

**Figure 3:**
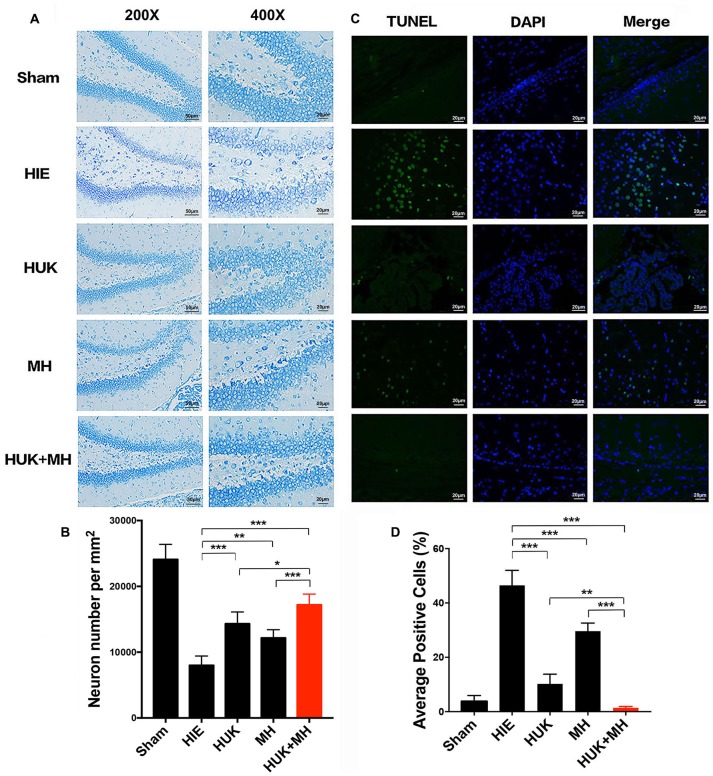
Nissl and TUNEL Staining of the right hippocampal region.** (A)** Nissl staining in hippocampus dentate gyrus (DG). Representative images of Niss-staining in the hippocampus DG are shown at 200× (the left images, Scale bar = 50 μm) and 400× magnification (the right images, Scale bar = 20 μm). Nissl bodies are deep blue and the nuclei are light blue. The most normal neurons are seen in the combination group compared to the HUK, MH and HIE groups (*n* = 3). **(B)** The Quantification of Nissl staining in hippocampus dentate gyrus. Each bar shows the average cell number per mm^2^ of each group. HUK had an interaction with MH (two-way ANOVA, *F* = 31.824, ***P < 0.001). HUK or MH treatments attenuated HIE-induced neuronal decline (HUK: 14320.000 ± 1797.776 cells/mm^2^, MH: 12160.000 ± 1252.198 cells/mm^2^, HIE: 8000.000 ± 1414.214 cells/mm^2^, two-way ANOVA, Tukey’s Test, ***P_HUK-HIE_ < 0.001, **P_MH-HIE_ = 0.003). The combination had a better effect on the survival of neurons, better than HUK or MH alone (Combination: 17200.000 ± 1624.808 cells/mm^2^, ***P_Combi-HIE_ < 0.001, *P_Combi-HUK_ = 0.041, ***P_Combi-MH_ < 0.001). **(C)** TUNEL staining in hippocampus DG. TUNEL positive cells (*n* = 3), including cells with apoptosis and necrosis, are green and the nuclei of cells are blue. The merged images were made by TUNEL and DAPI staining. The magnification is 400×. Scale bar = 20 μm. **(D)** The Quantification of TUNEL. The images were quantified and presented by the ratio of the number of TUNEL positive cells to the number of nuclei (DAPI). Each bar shows the percentage of the average positive ratio for each group. HUK synergistically interacts with MH on TUNEL (two-way ANOVA, *F* = 104.600, ***P < 0.001). HUK or MH alone reduces the TUNEL-labeled cells compared to HIE (two-way ANOVA-Tukey’s test, ***P_HUK-HIE_ < 0.001, ***P_MH-HIE_ < 0.001), and the combination is better than MH (two-way ANOVA-Tukey’s test, **P_Combi-HUK_ = 0.005, ***P_Combi-MH_ < 0.001). *P < 0.05, **P < 0.01, ***P < 0.001.

We used neuronal number per mm^2^ to measure the survival neurons in Nissl staining. HUK had an interaction with MH as shown by the Nissl staining results (two-way ANOVA, *F* = 31.824, *P* < 0.001). In the sham group, the survival neurons in the hippocampal DG were the highest (24080.000 ± 2287.357 cells/mm^2^, Figures 3A,B). HUK or MH treatments attenuated HIE-induced neuronal number decline (HUK: 14320.000 ± 1797.776 cells/mm^2^, MH: 12160.000 ± 1252.198 cells/mm^2^, HIE: 8000.000 ± 1414.214 cells/mm^2^, two-way ANOVA, Tukey’s Test, ***P_HUK-HIE_ < 0.001, **P_MH-HIE_ = 0.003, Figures 3A,B). The combination had a better effect on the survival of neurons, better than HUK or MH alone (Combination: 17200.000 ± 1624.808 cells/mm^2^, ***P_Combi-HIE_ < 0.001, *P_Combi-HUK_ = 0.041, ***P_Combi-MH_ < 0.001, Figures 3A,B).

HUK synergistically interacted with MH as shown by the TUNEL staining results (two-way ANOVA, *F* = 104.600, *P* < 0.001). Accordingly, HIE induced apoptosis from 2.772 ± 1.58% (Sham group) to 48.480 ± 5.532%, as measured by TUNEL staining. HUK or MH alone induced a significant reduction in apoptosis (green; HUK: 10.230 ± 3.554%, MH: 29.600 ± 3.003, Figures 3C,D) compared to the HIE group (two-way ANOVA, Tukey’s test, ***P_HUK-HIE_ < 0.001, ***P_MH-HIE_ < 0.001, Figures 3C,D), and had a further protective effect when combined (1.229 ± 0.603%), better than HUK or MH alone (two-way ANOVA, Tukey’s test, **P_Combi-HUK_ = 0.005, ***P_Combi-MH_ < 0.001, Figures 3C,D).

### HUK Upregulated VEGF-Labeled Cells, Tight Junctions and BDKRB1/B2, and the Combination of HUK and MH Enhanced the Effects of HUK or MH Alone

**Figure 4:**
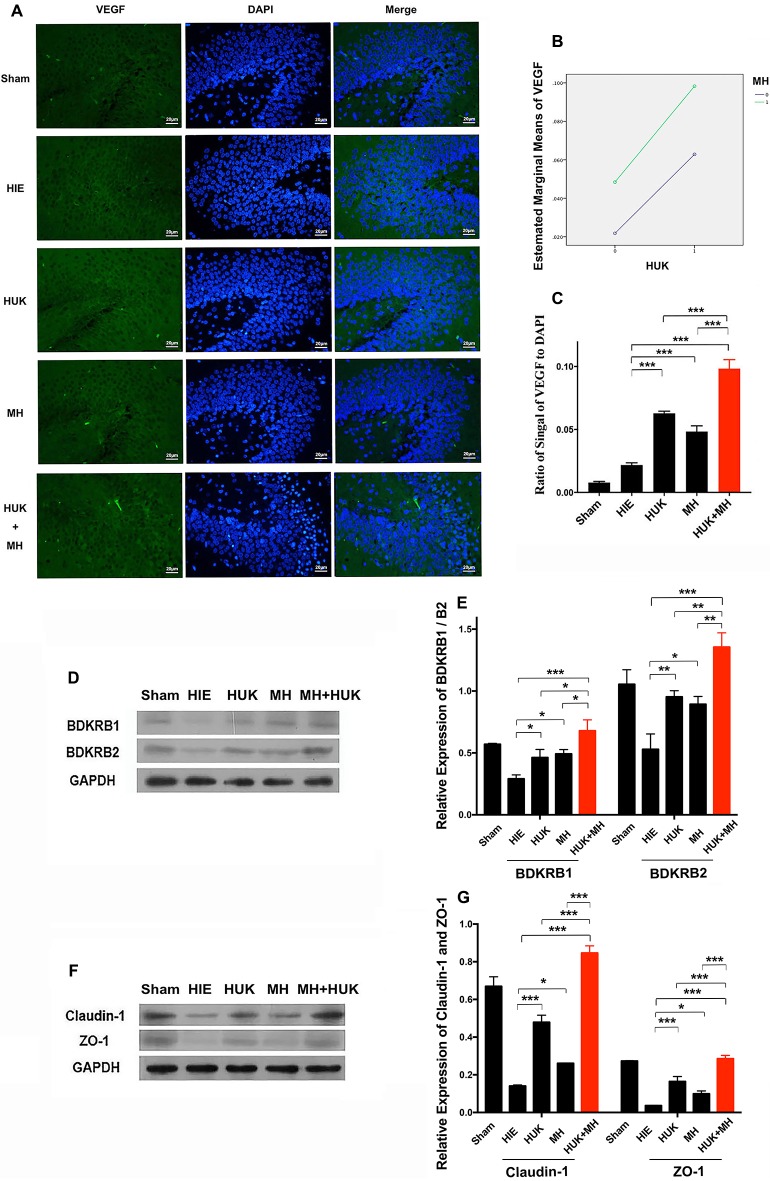
Comparison of vascular endothelial growth factor (VEGF)-labeled cells and Bradykinin B2/B1 receptor (BDKRB1/B2), Claudin-1 and Zonula Occludens (ZO)-1 proteins.** (A)** VEGF-labeled cells (*n* = 3): The VEGF-labeled cells are green and the nuclei are blue dyed by DAPI. The merged images were created by both anti-VEGF staining and DAPI staining. The magnification is 400×. Scale bar = 20 μm. **(B)** The interaction analysis of HUK and MH. HUK synergistically interacts with MH on VEGF-labeled cells (two-way ANOVA, *F* = 158.140, ***P < 0.001). **(C)** The quantification of VEGF. The images of VEGF-staining were quantified by the signal of VEGF normalized by the signal of nuclei. Each bar shows the average ratio of each group. HUK or MH alone increases the VEGF-staining compared with HIE (two-way ANOVA-Tukey’s test, ***P_HUK-HIE_ < 0.001, ***P_MH-HIE_ < 0.001), and the combination increases it to the peak (two-way ANOVA-Tukey’s test, ***P_Combi-HIE_ < 0.001), better than either of them alone (two-way ANOVA-Tukey’s test, ***P_Combi-HUK_ < 0.001, ***P_Combi-MH_ < 0.001). *P < 0.05, **P < 0.01, ***P < 0.001. **(D)** BDKRB1 and BDKRB2 expression lever. The detection of the level of BDKRB1 and BDKRB2 proteins (*n* = 3). GAPDH was used as an internal control protein (*n* = 3). **(E)** The quantification of BDKRB1 and BDKRB2. Each bar represents the ratio of the Gray value (GV) of each protein band normalized by the internal control protein GAPDH. HUK synergistically interacts with MH on BDKRB1 (two-way ANOVA, *F* = 21.053, *P* = 0.001) and BDKRB2 (two-way ANOVA, *F* = 38.759, *P* < 0.001). HUK or MH alone rescues the decrease in BDKRB1 caused by HIE (two-way ANOVA-Tukey’s test, *P_HUK-HIE_ = 0.036, *P_MH-HIE_ = 0.016), and the combination improves BDKRB1 compared to HIE (two-way ANOVA-Tukey’s test, ***P_Combi-HIE_ < 0.001) better than either HUK or MH (two-way ANOVA-Tukey’s test, *P_Combi-HUK_ = 0.010, *P_Combi-MH_ = 0.024). HUK or MH alone increases the BDKRB2 compared to HIE (two-way ANOVA-Tukey’s test, **P_HUK-HIE_ = 0.004, *P_MH-HIE_ = 0.012), and the combination gives the highest value (two-way ANOVA-Tukey’s test, ***P_Combi-HIE_ < 0.001), better than either HUK or MH (two-way ANOVA-Tukey’s test, **P_Combi-HUK_ = 0.007, **P_Combi-MH_ = 0.003). *P < 0.05, **P < 0.01, ***P < 0.001. **(F)** The expression of claudin-1 and ZO-1. The claudin-1 and ZO-1 proteins were measured by western blotting (*n* = 3). GAPDH was used as an internal control protein (*n* = 3). **(G)** The quantification of claudin-1 and ZO-1. Each bar shows the ratio of the GV of each protein band normalized to the internal control protein GAPDH. HUK and MH have synergistic interactions on claudin-1 and ZO-1 (two-way ANOVA, *F*_claudin-1_ = 395.489, ***P_Claudin-1_ < 0.001; *F*_ZO-1_ = 107.890, ***P_ZO-1_ < 0.001). HUK or MH increases the expression of claudin-1 (two-way ANOVA-Tukey’s test, ***P_HUK-HIE_ < 0.001, *P_MH-HIE_ = 0.012) and ZO-1 (two-way ANOVA-Tukey’s test, ***P_HUK-HIE_ < 0.001, *P_MH-HIE_ = 0.012), and combination increases claudin-1 and ZO-1 to the peak (two-way ANOVA-Tukey’s test, claudin-1: ***P_Combi-HIE_ < 0.001, ZO-1: ***P_Combi-HIE_ < 0.001), better than either of them alone (two-way ANOVA-Tukey’s test, claudin-1: ***P_Combi-HUK_ < 0.001, ***P_Combi-MH_ < 0.001; ZO-1: ***P_Combi-HUK_ < 0.001, ***P_Combi-MH_ < 0.001). *P < 0.05, **P < 0.01, ***P < 0.001.

Since HUK or combined treatment can prevent neural apoptosis, we investigated if HUK, MH or the combination can promote angiogenesis since vascular endothelial cells constitute of the critical part of BBB construction. The neuroprotective effect of HUK has been shown to be correlated with the BDKRB1 and the BDKRB2 (Han et al., 2015). We also examined if the protein levels of BDKRB1 and BDKRB2 were altered in the rat right hippocampus. Results showed that HUK had a synergistic effect with MH on VEGF-labeled cells (two-way ANOVA, *F* = 158.140, *P* < 0.001, Figure 4B). The VEGF-labeled cells were detected by anti-VEGF antibody immunofluorescent staining (VEGF, a marker of angiogenesis, mainly expressed in vascular endothelial cells, Beck and Plate, 2009). The ratios of the signal of VEGF-positive cell to cell nuclei (DAPI staining) increased in the HUK (0.063 ± 0.002) and MH groups (0.048 ± 0.004) compared to the HIE group (0.022 ± 0.002; two-way ANOVA, Tukey’s test, ***P_HUK-HIE_ < 0.001, ***P_MH-HIE_ < 0.001, Figures 4A,C). In the combined treatment group (0.098 ± 0.007), the ratio was higher than the other treatment groups (two-way ANOVA, Tukey’s test, ***P_Combi-HUK_ < 0.001, ***P_Combi-MH_ < 0.001, Figures 4A,C).

Furthermore, HUK synergistically interacts with MH on BDKRB1 (two-way ANOVA, *F* = 21.053, *P* = 0.001) and BDKRB2 (two-way ANOVA, *F* = 38.759, *P* < 0.001). HUK or MH obviously ameliorated the HIE-induced decrease in BDKRB1 (HUK: 0.463 ± 0.066, MH: 0.492 ± 0.035 vs. HIE: 0.291 ± 0.033, two-way ANOVA-Tukey’s test, *P_HUK-HIE_ = 0.036, *P_MH-HIE_ = 0.016, Figures 4D,E) and BDKRB2 proteins (HUK: 0.953 ± 0.050, MH: 0.893 ± 0.063 vs. HIE: 0.529 ± 0.124, two-way ANOVA-Tukey’s test, *P_HUK-HIE_ = 0.004, *P_MH-HIE_ = 0.012, Figures 4D,E). The combination of HUK and MH further markedly improved the HIE-induced decrease in BDKRB1 (0.679 ± 0.088) and BDKRB2 (1.355 ± 0.116, two-way ANOVA-Tukey’s test, B1 and B2: ***P_Combi-HIE_ < 0.001, Figures 4D,E), better than either HUK or MH (two-way ANOVA-Tukey’s test, B1: *P_Combi-HUK_ = 0.010, *P_Combi-MH_ = 0.024; B2: **P_Combi-HUK_ = 0.007, **P_Combi-MH_ = 0.003, Figures 4D,E).

We also found the same trends in the tight junction associated proteins claudin-1 and ZO-1 (Figure 4F). HUK synergistically interacted with MH to affect claudin-1 and ZO-1 (two-way ANOVA, *F*_Claudin-1_ = 395.489, *P*_Claudin-1_ < 0.001; *F*_ZO-1_ = 107.890, *P*_ZO-1_ < 0.001, Figures 4F,G). Claudin-1 and ZO-1, as measured by Western Blotting, obviously increased in the HUK (claudin-1: 0.479 ± 0.038, two-way ANOVA, Tukey’s test, ***P_HUK-HIE_ < 0.001; ZO-1: 0.164 ± 0.027, two-way ANOVA-Tukey’s test, ***P_HUK-HIE_ < 0.001, Figures 4F,G) and MH (claudin-1: 0.261 ± 0.002, two-way ANOVA, Tukey’s test, *P_MH-HIE_ = 0.012; ZO-1: 0.099 ± 0.016, *P_MH-HIE_ = 0.012, Figures 4F,G) groups and were the highest in the combination group (claudin-1: 0.846 ± 0.038, two-way ANOVA, Tukey’s test, ***P_Combi-HIE_ < 0.001; ZO-1: 0.285 ± 0.018, ***P_Combi-HIE_ < 0.001, Figures 4F,G) after HIE (claudin-1: 0.140 ± 0.006, ZO-1: 0.036 ± 0.002). The claudin-1 and ZO-1 in combination group were higher than HUK or HUK alone (two-way ANOVA-Tukey’s test, Claudin-1: ***P_Combi-HUK_ < 0.001, ***P_Combi-MH_ < 0.001; ZO-1: ***P_Combi-HUK_ < 0.001, ***P_Combi-MH_ < 0.001, Figures 4F,G). These data indicated that the neuroprotective effects of the combination came from the synergistic effects of HUK and MH on VEGF-labeled cells, claudin-1 and ZO-1 and were at least partly associated with BDKRB1 and BDKRB2.

### HUK Increased DCX-Labeled Cells and Cyclin D1, and the Combination of HUK and MH Enhanced These Effects

**Figure 5:**
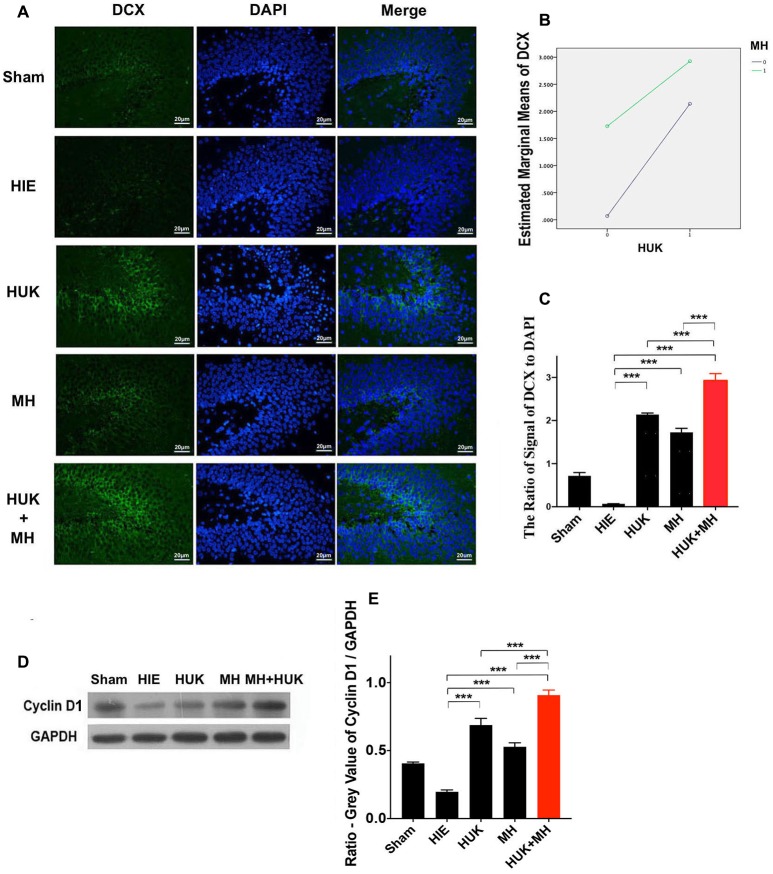
Comparison of Doublecortin (DCX)-labeled cells and the protein level of cyclin D1. **(A)** DCX staining (*n* = 3). The DCX-labeled cells are green and the nuclei are blue dyed by DAPI staining. The merged images were created by anti-DCX staining and DAPI staining. The magnification is 400×. Scale bar = 20 μm. **(B)** The interaction analysis of HUK and MH on DCX-staining. The HUK interacts with MH on DCX-staining (*F* = 404.200, ***P < 0.001). **(C)** The quantification of the DCX-staining. The images of DCX-staining were quantified by the signal of DCX normalized by the signal of nuclei. Each bar shows the average ratio of each group. Either HUK or MH increases the DCX-labeled cells after HIE (two-way ANOVA-Tukey’s test, ***P_HUK-HIE_ < 0.001, ***P_MH-HIE_ < 0.001). The combination increases it to the peak (two-way ANOVA-Tukey’s test, ***P_Combi-HIE_ < 0.001), better than HUK or MH alone (two-way ANOVA-Tukey’s test, ***P_Combi-HUK_ < 0.001, ***P_Combi-MH_ < 0.001). *P < 0.05, **P < 0.01, ***P < 0.001. **(D)** The expression of cyclin D1. The expression of cyclin D1 was measured by western blotting (*n* = 3). GAPDH was used as an internal control protein (*n* = 3). **(E)** The quantification of cyclin D1. Each bar represents the ratio of the GV of each protein band normalized to the internal control protein GAPDH. HUK additively interacts with MH on cyclin D1 (two-way ANOVA, *F* = 221.542, ***P < 0.001). HUK or MH alone increases the cyclin D1 level after HIE (two-way ANOVA-Tukey’s test, ***P_HUK-HIE_ < 0.001, ***P_MH-HIE_ < 0.001). The combination increases the cyclin D1 level to the highest value (two-way ANOVA- Tukey’s Test, ***P_Combi-HIE_ < 0.001), higher than HUK or MH alone (two-way ANOVA-Tukey’s test, ***P_Combi-HUK_ = 0.001, ***P_Combi-MH_ < 0.001). *P < 0.05, **P < 0.01, ***P < 0.001.

Given the enhancement effect of HUK and the combined treatment on angiogenesis, we further examined whether HUK and the combined treatment have a positive effect on neuronal regeneration (as marked by doublecortin, DCX) and cyclin D1, a regulator of the G1/S phase transition (Frade and Ovejero-Benito, 2015). Immunofluorescent staining showed an increase in the DCX-marked positive cells of the HUK or MH groups (HUK: 2.139 ± 0.036, MH: 1.725 ± 0.094 vs. HIE: 0.067 ± 0.004, two-way ANOVA, Tukey’s test, ***P_HUK-HIE_ < 0.001, ***P_MH-HIE_ < 0.001, Figures 5A,C). Although HUK had an additive interaction with MH on DCX-labeled positive cells (two-way ANOVA-Tukey’s test, *F* = 404.200, *P* < 0.001, Figure 5B), a stronger effect was observed in the combination group (2.926 ± 0.163, two-way ANOVA, Tukey’s test, ***P_Combi-HIE_ < 0.001, Figures 5A,C) than HUK or MH alone (two-way ANOVA, Tukey’s test, ***P_Combi-HUK_ < 0.001, ***P_Combi-MH_ < 0.001, Figures 5A,C).

Western blotting showed that HUK or MH attenuated the HIE-induced decrease in cyclin D1 (HUK: 0.688 ± 0.049, MH: 0.528 ± 0.030 vs. HIE: 0.197 ± 0.014, two-way ANOVA-Tukey’s test, ***P_HUK-HIE_ < 0.001, ***P_MH-HIE_ < 0.001, Figures 5D,E). HUK still had an additive interaction with MH on cyclin D1 (two-way ANOVA, *F* = 221.542, ***P < 0.001) and an enhanced protective effect was also seen for the combination treatment (2.926 ± 0.163, two-way ANOVA-Tukey’s test, ***P_Combi-HIE_ < 0.001, Figures 5D,E), higher than HUK or MH alone (two-way ANOVA-Tukey’s test, ***P_Combi-HUK_ < 0.001, ***P_Combi-MH_ < 0.001, Figures 5D,E), suggesting that the combination at least partly regulates the cell cycle to promote neurogenesis in HIE adult rats.

### HUK Increased Ki67-Labeled Cells, and the Combination of HUK and MH Enhanced the Effect

**Figure 6:**
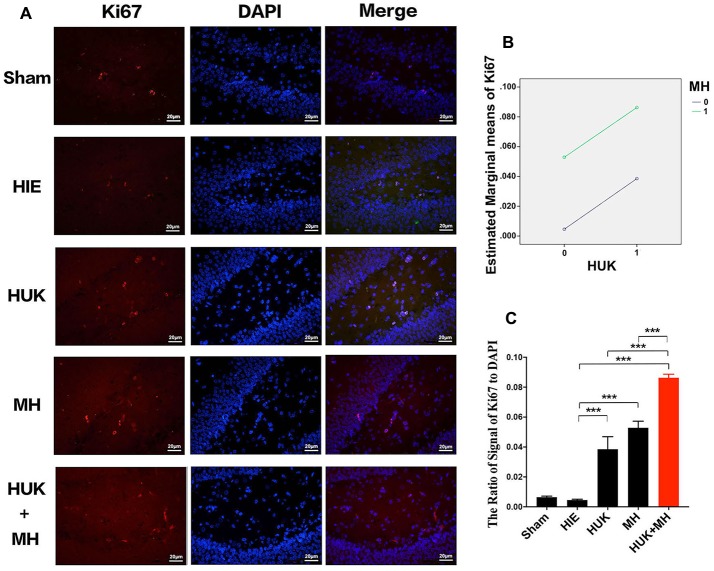
Comparison of the Ki67-labeled Cells. **(A)** Ki67 staining (*n* = 3). The Ki67-labeled cells are red and the nuclei are blue, as shown by DAPI staining. The merged images were created from the results of anti-Ki67 staining and DAPI staining. The magnification is 400×. Scale bar = 20 μm. **(B)** Interaction analysis of HUK and MH on Ki67 staining. HUK additively interacts with MH on Ki67 (two-way ANOVA, *F* = 178.700, ***P < 0.001). **(C)** The quantification of Ki67 staining. The images of Ki67-staining were quantified by the signal of Ki67 normalized by the signal of nuclei. Each bar shows the average ratio of each group. HUK or MH increases the Ki67-labeled cells (two-way ANOVA–Tukey’s test, ***P_HUK-HIE_ < 0.001, ***P_MH-HIE_ < 0.001). The combination increases the Ki67-positive cells to the highest (two-way ANOVA-Tukey’s test, ***P_Combi-HIE_ < 0.001), higher than HUK or MH alone (two-way ANOVA-Tukey’s test, ***P_Combi-HUK_ < 0.001, ***P_Combi-MH_ < 0.001). *P < 0.05, **P < 0.01, ***P < 0.001.

We used Ki67 staining to confirm the non-specific proliferation of all cells. Ki67 labels the G and S phase cells and highly expresses in proliferative cells. The results showed that HUK additively interacts with MH on Ki67-labeled cells (two-way ANOVA, *F* = 178.700, *P* < 0.001, Figure 6B). The signal of Ki67 staining normalized by the signal of DAPI had no significant change after HIE induced (0.005 ± 0.001), compared with sham group (0.006 ± 0.001). HUK or MH obviously increased the Ki67 to 0.039 ± 0.008 (two-way ANOVA, Tukey’s test, ***P_HUK-HIE_ < 0.001, Figures 6A,C) and (0.053 ± 0.004, two-way ANOVA, Tukey’s test, ***P_MH-HIE_ < 0.001, Figures 6A,C). Moreover, the combination increased the Ki67 to the highest level 0.086 ± 0.002 (two-way ANOVA, Tukey’s test, ***P_Combi-HIE_ < 0.001, Figures 6A,C), better than either HUK or MH alone (two-way ANOVA, Tukey’s test, ***P_Combi-HUK_ < 0.001, ***P_Combi-MH_ < 0.001, Figures 6A,C).

## Discussion

This study shows that HUK may be a potential neuroprotectant, functioning to induce angiogenesis and promote neuronal regeneration in an adult HIE rodent model. In this study, we investigated the effects of MH and HUK in the HIE model and noted four principal findings. First, we observed that the combination of HUK and MH rescued all adult HIE rats from death and profoundly improved neurological dysfunction at days 7. Second, MH significantly attenuated the tight-junction disruptions and increased the expressions of VEGF, BDKR B1/2, cyclin D1, Ki67 and doublecortin (DCX). Third, HUK enhanced these neuroprotective effects of MH. Lastly, the combination of MH and HUK may have their angiogenesis and neuronal regeneration effects at least partially via upregulating the expression of BDKR B1/2, and doublecortin (DCX). To our knowledge, this is the first study to explore the neuro-pathogenesis of combined MH and HUK therapy in the HIE model.

Although several lines of evidence demonstrate that HUK and MH may play important roles against ischemic stroke (Chen et al., 2010; Gao et al., 2014; Han et al., 2015), the efficacy of either single or combined treatment in HIE still needs to be explored and the neuro-pathogeneses involved in the neuroprotection need to be confirmed. From day 1 to 7, although neither HUK nor MH attenuated the neurological deficit, their combination profoundly ameliorated behavioral impairment, strongly suggesting that HUK enhanced the beneficial effect of MH. The neurological behavioral improvement continued for all observations on days 1, 3 and 7 after HIE, indicating that a short period of administration of exogenous HUK can persistently improve neurological dysfunction over a relatively long time. This result is similar to Han’s study showing that HUK could improve neurological dysfunction in ischemic stroke rats (Han et al., 2015). We found that HUK or MH prevented neurons from undergoing necrosis and apoptosis, while this combination led to a more pronounced neuronal survival, indicating that this combination of HUK and MH could provide more effective neuroprotection and this combination enhanced the neuroprotective effects of either alone.

It has been well documented that ZO-1 (Tight junction protein-1) and claudin-1, a group of integral membrane proteins, are closely associated with BBB integrity, representing one mode of cell-to-cell adhesion in endothelial cell sheets and responsible for the barrier function of brain vessels (Luissint et al., 2012; Ren et al., 2015; Li et al., 2016; Zanotto et al., 2017; Bhargavan and Kanmogne, 2018). Dysfunction of BBB mechanisms is well established after ischemia (Fernández-López et al., 2012; Oakley and Tharakan, 2014; Liu et al., 2017); however, we know less about how tight junctions respond to HIE insult and if HUK/MH influences the tight junctions under HIE stimulus. Our study showed that the expression of ZO-1 and claudin-1 in the cortex were significantly downregulated on day 2 following HIE insult and that MH attenuated this downregulation. This finding is consistent with previous studies indicating a similar BBB disruption with changes in tight-junction proteins in neonatal HIE and ischemic stroke (Ek et al., 2015; Shi et al., 2017). Our result strongly indicated that HIE insult may significantly disrupt the integrity of the BBB, while HUK or MH prevent the injury of endothelial cells and decrease the loss of tight junctions. In addition, the combination of HUK and MH exerted a much larger benefit on tight junction expression in HIE rats, and this attenuation of the destruction of tight junctions may contribute to the protective effects of HUK and MH on BBB integrity. These findings (Figures 4A–C,F,G) suggest that the synergistic neuroprotective effects of HUK and MH on BBB may at least partially result from endothelial cell stabilization and decreases in the loss of tight junctions.

Accumulated evidence has shown that HUK may be associated with angiogenesis in ischemic stroke (Ek et al., 2015). We propose that in HIE rats, HUK may exert neuroprotection partially via modulating angiogenesis and regeneration. To investigate this hypothesis, we detected VEGF, BDKR B1/B2, Doublecortin (DCX), Cyclin D and Antigen Ki-67 (Ki-67) along the hippocampus by immunohistochemistry and western blotting.

In the current study, the increased VEGF at day 2 following HIE along the hippocampus suggests that angiogenesis may occur after HIE onset. This result is similar to previous studies (Schoch et al., 2002; Han et al., 2015; Shi et al., 2017), indicating that hypoxic insult may lead to a significant increase in the levels of VEGF in mouse brain that correlated with the hypoxic stimulus. This upregulated VEGF expression induced by HIE in the brain may play both neurotoxic and neuroprotective roles: (1) leading to vasogenic edema and the leakage of blood-borne substances into the brain parenchyma and increased BBB permeability; and (2) stimulating and sustaining regeneration and neovascularization. The neuroprotective effect of upregulated VEGF occurs from several hours to several weeks following hypoxic-ischemia (Sun and Guo, 2005; Kaur and Ling, 2009; Baburamani et al., 2012). Since VEGF has been well documented as playing a role in angiogenesis (Doeppner et al., 2017; Zhao et al., 2017; Zou et al., 2017), our finding demonstrates that either HUK or MH promotes angiogenesis, but the combination gave the highest value. In addition, we also noted the downregulated expression of the BDKRB1/2 after HIE insult, while HUK, MH and the combination restored the downregulation of BDKRB1/2. It has been documented that the activation of BDKRB1/2 is involved in the angiogenesis process (Spinetti et al., 2011; Mousa et al., 2014). Our observation that HUK mediated angiogenesis following HIE insult is similar to previous studies (Han et al., 2015; Li et al., 2015). Xu’s group found that in ischemic experimental models and stroke patients, HUK promoted post-ischemic angiogenesis and cerebral perfusion via increasing vessel density, enhancing VEGF and apelin/APJ expression, and inducing ERK1/2 phosphorylation (Han et al., 2015; Li et al., 2015). Taken together, these data strongly indicate that the BDKRB1/B2 receptors were involved in the angiogenetic effect of HUK in HIE rats, and HUK may promote angiogenesis in HIE stimulus via upregulating the expression of VEGF and BDKRB1/2.

To further explore whether the combined effects of HUK and MH on the HIE insult were associated with neurogenesis or regeneration of all kinds of cells, DCX, which is an important predictor of neurogenesis, and cyclin D1 and Ki-67, which are predictors of non-specific regeneration, were evaluated. Cyclin D1 is a member of the cyclin protein family, plays essential roles in regulating cell cycle progression and proliferation and is usually recognized as a biomarker of regeneration of cells (Bouchard-Cannon et al., 2013; Cheyuo et al., 2015). Ki67 labels the G and S phase cells and highly expresses in proliferative cells (Yang et al., 2018). In the current study, our observation that cyclin D1 and Ki67 decreased with adult HIE insult implies that hypoxic-ischemic cells undergo death and regeneration is markedly reduced. Impressively, either MH or HUK treatment significantly reversed the HIE-induced downregulation of cyclin D1 and Ki67, strongly indicating that MH (Yenari and Han, 2013; Wood et al., 2016) or HUK following HIE onset could enhance regeneration by positively influencing regeneration of neurons or other cell. The mechanisms of MH or HUK enhancing regenerative properties in the current study could be explained, in part, by its effect on reducing apoptosis (Figure 3) and enhancing angiogenesis in a crucial time window, as indicated by the upregulation of VEGF. This MH-induced neurogenesis in the current work is consistent with Silasi and Colbourne’s (2011) study, indicating that hypothermia promoted CA neurogenesis and cell regeneration in global ischemia. To further identify the effects of MH and HUK on neurogenesis, we examined the expression of doublecortin (DCX), a candidate marker for adult neurogenesis, by immunohistochemical staining in the hippocampus, and observed a change similar to cyclin D1 (Figures 5D,E). More interestingly, our study showed that the combination of MH and HUK led to more pronounced increase in the expression of DCX when compared to MH or HUK alone, indicating greater efficacies of neurogenesis. To our knowledge, this is the first study showing that HUK exerts neurogenesis functions by regulating DCX in adult HIE insult.

In addition to mediating angiogenesis and promoting proliferation, other mechanisms, like inhibiting vasodilation, suppressing inflammation and activating the innate immune response, are found to be involved in HUK’s effects against ischemia (Ponticelli and Meroni, 2009; Chen et al., 2010; Sharma and Narayanan, 2014). In addition, Chen et al. (2010) found that HUK not only suppresses cerebral inflammation and downregulates NF-κB, but also activates the MAPK/ERK pathway in an experimental ischemic model. Whether those molecular mechanisms play important roles in HIE insult needs to be further explored.

In summary, for the first time, this study demonstrates the beneficial effects of a combination therapy of MH and HUK for treating adult HIE rats. The combination of MH and HUK was associated with improvement in functional recovery, combined cellular neuroprotection, attenuation of tight junction reduction, promotion of angiogenesis and regeneration in HIE rats. This finding is clinically meaningful because it strongly implies that an early combination of MH and HUK may correlate with improved outcomes and reduced mortality in patients with HIE. These data support the strategy of combining MH with pharmacological HUK to increase the clinical feasibility, efficacy and safety of the treatment of HIE patients. The combined treatment could represent a promising approach to create more effective and safer hypothermia therapies for patients with brain injuries.

## Author Contributions

XG, HX, SZ, QW and YT: conceived and designed the study. XG and HX: performed the study. QO, YJ, PW and YX: analyzed the data. BY, CW and XY: contributed reagents, materials, and analysis tools. XG, QW and YT: wrote the article.

## Conflict of Interest Statement

The authors declare that the research was conducted in the absence of any commercial or financial relationships that could be construed as a potential conflict of interest.

## Abbreviations

BBB: Blood-brain barrier
BDKRB1: Bradykinin receptor B1
BDKRB2: Bradykinin receptor B2
DCX: Doublecortex
ELAM-1: selectin E
HIE: Hypoxic-ischemic encephalopathy
HUK: Human urinary kallidinogenase
IL-1: Interleukin-1
IL-6: Interleukin-6
LOX-1: low-density lipoprotein receptor-1
MCP-1: Monocyte chemoattractant protein 1
MH: Mild hypothermia
NF-κB: Nuclear Factor-κB
nNOS: nitric oxide synthase
OGD/R: Oxygen and glucose deprivation-reoxgenation
TNF-*α*: Tumor necrosis factor-*α*
TUNEL: Terminal deoxynucleotidyl transferase-mediated dUTP nick end-labeling
VEGF: Vascular endothelial growth factor
ZO-1: Zonula occludens-1.

## References

[B1] Akamatsu T. Dai H. Mizuguchi M. Goto Y. Oka A. Itoh M. (2014). LOX-1 is a novel therapeutic target in neonatal hypoxic-ischemic encephalopathy. Am. J. Pathol. 184, 1843–1852. 10.1016/j.ajpath.2014.02.022 24731447

[B2] Baburamani A. A. Ek C. J. Walker D. W. Castillo-melendez M. (2012). Vulnerability of the developing brain to hypoxic-ischemic damage: contribution of the cerebral vasculature to injury and repair? Front. Physiol. 3:424. 10.3389/fphys.2012.00424 23162470 PMC3493883

[B3] Beck H. Plate K. H. (2009). Angiogenesis after cerebral ischemia. Acta Neuropathol. 117, 481–496. 10.1007/s00401-009-0483-6 19142647

[B4] Bhargavan B. Kanmogne G. D. (2018). Differential mechanisms of inflammation and endothelial dysfunction by HIV-1 subtype-B and recombinant CRF02_AG tat proteins on human brain microvascular endothelial cells: implications for viral neuropathogenesis. Mol. Neurobiol. 55, 1352–1363. 10.1007/s12035-017-0382-0 28127697 PMC6507400

[B5] Bouchard-Cannon P. Mendoza-Viveros L. Yuen A. Kærn M. Cheng H.-Y. M. (2013). The circadian molecular clock regulates adult hippocampal neurogenesis by controlling the timing of cell-cycle entry and exit. Cell Rep. 5, 961–973. 10.1016/j.celrep.2013.10.037 24268780

[B6] Chao J. Chao L. (2006). Experimental therapy with tissue kallikrein against cerebral ischemia. Front. Biosci. 11, 1323–1327. 10.2741/1886 16368519

[B7] Chao J. Shen B. Gao L. Xia C.-F. Bledsoe G. Chao L. (2010). Tissue kallikrein in cardiovascular, cerebrovascular and renal diseases and skin wound healing. Biol. Chem. 391, 345–355. 10.1515/BC.2010.042 20180644

[B8] Chen Z. Huang D. Niu F. Zhang X. Li E. Xu Y. (2010). Human urinary kallidinogenase suppresses cerebral inflammation in experimental stroke and downregulates nuclear factor-κB. J. Cereb. Blood Flow Metab. 30, 1356–1365. 10.1038/jcbfm.2010.19 20179726 PMC2949229

[B9] Cheyuo C. Aziz M. Yang W.-L. Jacob A. Zhou M. Wang P. (2015). Milk fat globule-EGF factor VIII attenuates CNS injury by promoting neural stem cell proliferation and migration after cerebral ischemia. PLoS One 10:e0122833. 10.1371/journal.pone.0122833 25867181 PMC4394995

[B10] Couillard-Despres S. Winner B. Schaubeck S. Aigner R. Vroemen M. Weidner N.. (2005). Doublecortin expression levels in adult brain reflect neurogenesis. Eur. J. Neurosci. 21, 1–14. 10.1111/j.1460-9568.2004.03813.x 15654838

[B12] Doeppner T. R. Traut V. Heidenreich A. Kaltwasser B. Bosche B. Bähr M.. (2017). Conditioned medium derived from neural progenitor cells induces long-term post-ischemic neuroprotection, sustained neurological recovery, neurogenesis and angiogenesis. Mol. Neurobiol. 54, 1531–1540. 10.1007/s12035-016-9748-y 26860410

[B13] Edwards A. B. Feindel K. W. Cross J. L. Anderton R. S. Clark V. W. Knuckey N. W.. (2017). Modification to the rice-vannucci perinatal hypoxic-ischaemic encephalopathy model in the P7 rat improves the reliability of cerebral infarct development after 48 hours. J. Neurosci. Methods 288, 62–71. 10.1016/j.jneumeth.2017.06.016 28648719

[B14] Ek C. J. D’Angelo B. Baburamani A. A. Lehner C. Leverin A.-L. Smith P. L. P.. (2015). Brain barrier properties and cerebral blood flow in neonatal mice exposed to cerebral hypoxia-ischemia. J. Cereb. Blood Flow Metab. 35, 818–827. 10.1038/jcbfm.2014.255 25627141 PMC4420855

[B15] Fernández-López D. Faustino J. Daneman R. Zhou L. Lee S. Y. Derugin N.. (2012). Blood-brain barrier permeability is increased after acute adult stroke but not neonatal stroke in the rat. J. Neurosci. 32, 9588–9600. 10.1523/JNEUROSCI.5977-11.2012 22787045 PMC3539825

[B16] Frade J. M. Ovejero-Benito M. C. (2015). Neuronal cell cycle: the neuron itself and its circumstances. Cell Cycle 14, 712–720. 10.1080/15384101.2015.1004937 25590687 PMC4418291

[B17] Gao X.-Y. Huang J.-O. Hu Y.-F. Gu Y. Zhu S.-Z. Huang K.-B.. (2014). Combination of mild hypothermia with neuroprotectants has greater neuroprotective effects during oxygen-glucose deprivation and reoxygenation-mediated neuronal injury. Sci. Rep. 4:7091. 10.1038/srep07091 25404538 PMC4665348

[B18] Gao X.-Y. Zhu S.-Z. Xiang W. Huang K.-B. Hu Y.-F. Gu Y.. (2016). Prolonged hypothermia exposure diminishes neuroprotection for severe ischemic-hypoxic primary neurons. Cryobiology 72, 141–147. 10.1016/j.cryobiol.2016.01.003 26802735

[B19] Garcia K. O. Ornellas F. L. M. Martin P. K. M. Patti C. L. Mello L. E. Frussa-Filho R.. (2014). Therapeutic effects of the transplantation of VEGF overexpressing bone marrow mesenchymal stem cells in the hippocampus of murine model of Alzheimer’s disease. Front. Aging Neurosci. 6:30. 10.3389/fnagi.2014.00030 24639647 PMC3945612

[B20] Guardia Clausi M. Paez P. M. Pasquini L. A. Pasquini J. M. (2016). Inhalation of growth factors and apo-transferrin to protect and repair the hypoxic-ischemic brain. Pharmacol. Res. 109, 81–85. 10.1016/j.phrs.2016.01.010 26804249

[B21] Han L. Li J. J. Chen Y. Zhang M. Qian L. Chen Y.. (2015). Human urinary kallidinogenase promotes angiogenesis and cerebral perfusion in experimental stroke. PLoS One 10:e0134543. 10.1371/journal.pone.0134543 26222055 PMC4519127

[B22] Harding B. Conception K. Li Y. Zhang L. (2016). Glucocorticoids protect neonatal rat brain in model of hypoxic-ischemic encephalopathy (HIE). Int. J. Mol. Sci. 18:E17. 10.3390/ijms18010017 28025500 PMC5297652

[B23] Kaur C. Ling E. A. (2009). Periventricular white matter damage in the hypoxic neonatal brain: role of microglial cells. Prog. Neurobiol. 87, 264–280. 10.1016/j.pneurobio.2009.01.003 19428957

[B24] Kossatz E. Maldonado R. Robledo P. (2016). CB2 cannabinoid receptors modulate HIF-1α and TIM-3 expression in a hypoxia-ischemia mouse model. Eur. Neuropsychopharmacol. 26, 1972–1988. 10.1016/j.euroneuro.2016.10.003 28253997

[B25] Lee B. S. Jung E. Lee Y. Chung S.-H. (2017). Hypothermia decreased the expression of heat shock proteins in neonatal rat model of hypoxic ischemic encephalopathy. Cell Stress Chaperones 22, 409–415. 10.1007/s12192-017-0782-0 28285429 PMC5425372

[B26] Li J. Chen Y. Zhang X. Zhang B. Zhang M. Xu Y. (2015). Human urinary kallidinogenase improves outcome of stroke patients by shortening mean transit time of perfusion magnetic resonance imaging. J. Stroke Cerebrovasc. Dis. 24, 1730–1737. 10.1016/j.jstrokecerebrovasdis.2015.03.032 26139453

[B27] Li Z. Mo N. Li L. Cao Y. Wang W. Liang Y.. (2016). Surgery-induced hippocampal angiotensin II elevation causes blood-brain barrier disruption via MMP/TIMP in aged rats. Front. Cell. Neurosci. 10:105. 10.3389/fncel.2016.00105 27199659 PMC4844612

[B28] Liu N. Chen H. Wu B. Li Y. Wintermark M. Jackson A.. (2017). CT permeability imaging predicts clinical outcomes in acute ischemic stroke patients treated with intra-arterial thrombolytic therapy. Mol. Neurobiol. 54, 2539–2546. 10.1007/s12035-016-9838-x 26988262

[B29] Luissint A.-C. Artus C. Glacial F. Ganeshamoorthy K. Couraud P.-O. (2012). Tight junctions at the blood brain barrier: physiological architecture and disease-associated dysregulation. Fluids Barriers CNS 9:23. 10.1186/2045-8118-9-23 23140302 PMC3542074

[B30] Manley B. J. Owen L. S. Hooper S. B. Jacobs S. E. Cheong J. L. Y. Doyle L. W.. (2017). Towards evidence-based resuscitation of the newborn infant. Lancet 389, 1639–1648. 10.1016/S0140-6736(17)30547-0 28443558

[B31] Miao J. Deng F. Zhang Y. Xie H. Feng J. (2016). Exogenous human urinary kallidinogenase increases cerebral blood flow in patients with acute ischemic stroke. Neurosciences 21, 126–130. 10.17712/nsj.2016.2.20150581 27094522 PMC5107266

[B32] Mitra S. Bale G. Highton D. Gunny R. Uria-Avellanal C. Bainbridge A.. (2017). Pressure passivity of cerebral mitochondrial metabolism is associated with poor outcome following perinatal hypoxic ischemic brain injury. J. Cereb. Blood Flow Metab. [Epub ahead of print]. 10.1177/0271678x17733639 28949271 PMC6311664

[B33] Mousa S. A. Lin H.-Y. Tang H. Y. Hercbergs A. Luidens M. K. Davis P. J. (2014). Modulation of angiogenesis by thyroid hormone and hormone analogues: implications for cancer management. Angiogenesis 17, 463–469. 10.1007/s10456-014-9418-5 24458693

[B34] Ni J. Qu J. Yao M. Zhang Z. Zhong X. Cui L.. (2017). Re-evaluate the efficacy and safety of human urinary kallidinogenase (RESK): protocol for an open-label, single-arm, multicenter phase IV trial for the treatment of acute ischemic stroke in Chinese patients. Transl. Stroke Res. 8, 341–346. 10.1007/s12975-017-0527-5 28265861

[B35] Oakley R. Tharakan B. (2014). Vascular hyperpermeability and aging. Aging Dis. 5, 114–125. 10.14336/AD.2014.0500114 24729937 PMC3966670

[B36] Ponticelli C. Meroni P. L. (2009). Kallikreins and lupus nephritis. J. Clin. Invest. 119, 768–771. 10.1172/jci38786 19348047 PMC2662572

[B37] Ren C. Li N. Wang B. Yang Y. Gao J. Li S.. (2015). Limb ischemic perconditioning attenuates blood-brain barrier disruption by inhibiting activity of MMP-9 and occludin degradation after focal cerebral ischemia. Aging Dis. 6, 406–417. 10.14336/AD.2015.0812 26618042 PMC4657812

[B38] Schoch H. J. Fischer S. Marti H. H. (2002). Hypoxia-induced vascular endothelial growth factor expression causes vascular leakage in the brain. Brain 125, 2549–2557. 10.1093/brain/awf257 12390979

[B11] Shankaran S. Pappas A. McDonald S. A. Vohr B. R. Hintz S. R. Yolton K.. (2012). Childhood outcomes after hypothermia for neonatal encephalopathy. N. Engl. J. Med. 366, 2085–2092. 10.1056/NEJMoa1112066 22646631 PMC3459579

[B39] Sharma J. N. Narayanan P. (2014). The kallikrein-kinin pathways in hypertension and diabetes. Prog. Drug Res. 69, 15–36. 10.1007/978-3-319-06683-7_2 25130038

[B40] Shi S. Qi Z. Ma Q. Pan R. Timmins G. S. Zhao Y.. (2017). Normobaric hyperoxia reduces blood occludin fragments in rats and patients with acute ischemic stroke. Stroke 48, 2848–2854. 10.1161/STROKEAHA.117.017713 28931617 PMC5659343

[B41] Silasi G. Colbourne F. (2011). Therapeutic hypothermia influences cell genesis and survival in the rat hippocampus following global ischemia. J. Cereb. Blood Flow Metab. 31, 1725–1735. 10.1038/jcbfm.2011.25 21364603 PMC3170941

[B42] Spinetti G. Fortunato O. Cordella D. Portararo P. Krankel N. Katare R.. (2011). Tissue kallikrein is essential for invasive capacity of circulating proangiogenic cells. Circ. Res. 108, 284–293. 10.1161/CIRCRESAHA.110.236786 21164105 PMC3596779

[B43] Stankowski J. N. Gupta R. (2011). Therapeutic targets for neuroprotection in acute ischemic stroke: lost in translation?. Antioxid. Redox Signal. 14, 1841–1851. 10.1089/ars.2010.3292 20626319 PMC3120088

[B44] Sun F.-Y. Guo X. (2005). Molecular and cellular mechanisms of neuroprotection by vascular endothelial growth factor. J. Neurosci. Res. 79, 180–184. 10.1002/jnr.20321 15573409

[B45] Thornton C. Leaw B. Mallard C. Nair S. Jinnai M. Hagberg H. (2017). Cell death in the developing brain after hypoxia-ischemia. Front. Cell. Neurosci. 11:248. 10.3389/fncel.2017.00248 28878624 PMC5572386

[B46] Wood T. Osredkar D. Puchades M. Maes E. Falck M. Flatebø T.. (2016). Treatment temperature and insult severity influence the neuroprotective effects of therapeutic hypothermia. Sci. Rep. 6:23430. 10.1038/srep23430 26997257 PMC4800445

[B47] Yang L. Tucker D. Dong Y. Wu C. Lu Y. Li Y.. (2018). Photobiomodulation therapy promotes neurogenesis by improving post-stroke local microenvironment and stimulating neuroprogenitor cells. Exp. Neurol. 299, 86–96. 10.1016/j.expneurol.2017.10.013 29056360 PMC5723531

[B48] Yang W. Zhang X. Wang N. Tan J. Fang X. Wang Q.. (2016). Effects of acute systemic hypoxia and hypercapnia on brain damage in a rat model of hypoxia-ischemia. PLoS One 11:e0167359. 10.1371/journal.pone.0167359 27907083 PMC5131999

[B49] Yenari M. A. Han H. S. (2013). Influence of therapeutic hypothermia on regeneration after cerebral ischemia. Front. Neurol. Neurosci. 32, 122–128. 10.1159/000346428 23859971 PMC4022181

[B50] Zanotto C. Simão F. Gasparin M. S. Biasibetti R. Tortorelli L. S. Nardin P.. (2017). Exendin-4 reverses biochemical and functional alterations in the blood-brain and blood-CSF barriers in diabetic rats. Mol. Neurobiol. 54, 2154–2166. 10.1007/s12035-016-9798-1 26927659

[B51] Zhang C. Tao W. Liu M. Wang D. (2012). Efficacy and safety of human urinary kallidinogenase injection for acute ischemic stroke: A systematic review. J. Evid. Based Med. 5, 31–39. 10.1111/j.1756-5391.2012.01167.x 23528118

[B52] Zhao G. Cheng X. W. Piao L. Hu L. Lei Y. Yang G.. (2017). The soluble VEGF receptor sFlt-1 contributes to impaired neovascularization in aged mice. Aging Dis. 8, 287–300. 10.14336/AD.2016.0920 28580185 PMC5440109

[B53] Zou J. Chen Z. Wei X. Chen Z. Fu Y. Yang X.. (2017). Cystatin C as a potential therapeutic mediator against Parkinson’s disease via VEGF-induced angiogenesis and enhanced neuronal autophagy in neurovascular units. Cell Death Dis. 8:e2854. 10.1038/cddis.2017.240 28569795 PMC5520899

